# Iopofosine I-131 treatment in late-line patients with relapsed/refractory multiple myeloma post anti-BCMA immunotherapy

**DOI:** 10.1038/s41408-022-00725-2

**Published:** 2022-09-06

**Authors:** Jarrod Longcor, Natalie Callander, Kate Oliver, Asher Chanan-Khan, Sikander Ailawadhi

**Affiliations:** 1 Cellectar Biosciences, Inc., Florham Park, NJ USA; 2 University of Wisconsin Carbone Cancer Center, Madison, WI USA; 3 Division of Hematology-Oncology, Mayo Clinic, Jacksonville, FL USA; 4 Department of Cancer Biology, Mayo Clinic, Jacksonville, FL USA

**Keywords:** Medical research, Health care

Dear Editor,

Multiple myeloma (MM) is the second most common hematologic malignancy, accounting for 10% of all hematologic cancers [1]. The disease is characterized by the uncontrolled expansion of malignant plasma cells in the bone marrow, resulting in aberrantly high levels of monoclonal immunoglobulins that can be detected in the blood, urine, or both. Although effective pharmacologic therapy and autologous hematopoietic stem cell transplantation have improved patient response rates and progression-free survival (PFS) [1, 2], the prognosis for patients with multi-drug refractory MM remains poor [2, 3]. B-cell maturation antigen (BCMA), a transmembrane glycoprotein in the tumor necrosis factor receptor superfamily 17 (*TNFRSF17*), is highly expressed by plasma cells, and can be effectively targeted via chimeric antigen receptor (CAR) T cells [4]. Recent studies demonstrate significant clinical responses in patients with relapsed/refractory MM (R/RMM) despite having failed multiple prior treatments (including proteasome inhibitors [PIs] and immunomodulatory drugs [IMiDs]) [5, 6]. Two BCMA-directed commercially available CAR T products have been approved and their use is expected to grow exponentially. Despite the success of these BCMA-targeting agents in MM, none are curative and some patients treated with BCMA continue to have an aggressive clinical course and a poor prognosis. At present, there are limited options for patients with MM who are triple-class refractory (PI, IMiDs, and anti-CD38 monoclonal antibody) and relapsed or refractory to anti-BCMA immunotherapies. The increasing use of anti-CD38 monoclonal antibodies in combination with IMiDs and PIs in newly diagnosed patients is limiting later-line options. This highly refractory patient population represents an unmet clinical need with limited options. Iopofosine I-131 (iopofosine [formerly known as CLR 131, iodine-131 [I-131]-CLR-1404]) is a first-in-class phospholipid radioconjugate in which I-131 is covalently bound to a phospholipid ether (PLE) mimetic engineered to target lipid rafts in tumor cells [7]. Upon binding to the lipid rafts, iopofosine is internalized, delivering I-131 intracellularly and causing double-stranded DNA breaks via the β-emission from I-131, resulting in apoptosis. Additionally, the PLE portion of the molecule inhibits AKT. Phase I trials with iopofosine in patients with advanced solid tumors or highly pretreated R/RMM have shown that iopofosine can be safely administered intravenously and elicits a deep and durable response in highly pretreated populations [8, 9]. In patients with triple-class refractory R/RMM receiving a total dose of >60 mCi, an overall response rate (ORR) of 40% and PFS of >3 months have been observed in early trials.

The present study reports on an expansion cohort of the ongoing CLOVER-1 study of iopofosine in relapsed/refractory B-cell populations. Patients in the study met the definition of triple-class refractory R/RMM and also failed or are refractory to anti-BCMA therapies (quad-class refractory). Patients had a median of 9 prior lines (4–17) of therapy. The study (NCT02952508) was reviewed and approved by the Institutional Review Board of each participating center. All patients signed informed consent before enrollment.

Adult patients with R/RMM were eligible if they had measurable disease by either M protein or serum free-light chains, adequate bone marrow, renal, hepatic, and coagulation function, and Eastern Cooperative Oncology Group Performance Status 0–2. Important exclusion criteria included previous total or hemi-body irradiation or external beam radiation resulting in >20% of total bone marrow receiving >20 Grays (Gys).

Study participants received a fractionated dose of 30 mCi/m^2^ iopofosine (15 mCi/m^2^ on day 1, and 15 ± 1 day) with an optional second cycle of iopofosine in the same split-dose regimen, at the discretion of the investigator. Participants also received low-dose dexamethasone (40 mg by mouth weekly for ≤12 weeks) and thyroid protection (starting 24 h before first infusion and continued for 14 days after the last dose in each cycle). Data was collected over 85 days following iopofosine infusion, with ongoing monitoring beyond 85 days. Response to treatment was evaluated using the International Myeloma Working Group Uniform Response Criteria for Multiple Myeloma [10].

Seven patients were enrolled; baseline characteristics are shown in Table 1. One patient received a total dose <60 mCi and continued to have progressive disease at the first assessment. Six participants received ≥60 mCi (mean total dose of 72.86 mCi); in this population ORR was 50%, with best response of stable disease (*n* = 3) or partial response (*n* = 3) (Table 1). Clinical benefit (stable disease or better) was 100% with the minor response rate (minimal response [>25% reduction in M protein] or better of 83.3%. At the time of data cutoff, median overall survival (OS) had not been reached, the mean OS was 9.1 months (2.6–22.4 months) in patients receiving ≥ 60 mCi total administered dose. Median PFS was 3 months (2.2–5 months).

**Table 1:** Baseline characteristics and efficacy measures of patients included in this analysis.

	All patients (*n* = 7)	≥60 mCi total administered dose (*n* = 6)
Median age (range)	63 (46–77)	68 (46–77)
Male:Female	5:2	5:1
Race, *n* (%)		
White or White European	6 (86)	5 (83)
Black or African American	1 (14)	1 (17)
Median prior therapies, *n* (range)	9 (4*–*17)	9 (4*–*17)
≤5 prior lines, *n* (%)	1 (14)	1 (17)
>5 prior lines, *n* (%)	6 (86)	5 (83.3)
Prior stem cell transplant, *n* (%)	7 (100)	6 (100)
ISS Disease Stage (at entry), *n* (%)		
Stage I	2 (29)	2 (33)
Stage II	2 (29)	2 (33)
Stage III	2 (29)	1 (17)
Unknown	1 (14)	1 (17)
Cytogenetic risk, *n* (%) Standard/High/Unknown	3 (43)/3 (43)/1 (14)	3 (50)/2 (33)/1 (17)
Triple-class refractory, *n* (%)	7 (100)	6 (100)
Prior anti-BCMA immunotherapy, *n* (%)		
CAR T-cell	2 (29)	2 (33)
Antibody drug conjugate	5 (71)	4 (67)
Median time from last anti-BCMA treatment	85 days (12–654)	97 days (12–654)
Efficacy measures for patients achieving ≥60 mCi total administered dose (*n* = 6) *n* (%)		
Overall response rate (PR or better)	3 (50)	
Clinical benefit (MR or better)	5 (83.3)	
Disease control (Stable disease or better)	6 (100)	

*ISS* International Staging System, *BCMA* B-cell maturation antigen, *CAR* chimeric antigen receptor, *MR* minimal response, *PR* partial response.

All patients experienced ≥1 adverse event (Table 2). No dosing delays, dose reductions, or treatment discontinuations were caused by AEs. The most common grade 3/4 AEs were cytopenias (thrombocytopenia 5 [75%] and neutropenia 4 [57%] of 7). None of the patients experienced febrile neutropenia. The 2 patients who experienced grade 4 neutropenia received a single injection of growth factor support. None of the patients experienced bleeding or infections associated with thrombocytopenia or neutropenia, respectively. All cytopenias resolved within the study period. None of the patients experienced any grade neuropathy, cardiopulmonary toxicity, renal or hepatic toxicities, ocular toxicities, cytokine release syndrome, or similarly related toxicities.

**Table 2:** Clinical response and treatment-emergent adverse events.

System organ class preferred term	All grades *n* (%)	Grade 1, 2 *n* (%)	Grade 3 *n* (%)	Grade 4 *n* (%)
Anemia	4 (57)	1 (14)	3 (43)	0 (0)
Leukopenia	4 (57)	1 (14)	0 (0)	3 (43)
Lymphopenia	2 (29)	0 (0)	1 (14)	1 (14)
Neutropenia	4 (57)	0 (0)	2 (29)	2 (29)
Thombocytopenia	5 (71)	0 (0)	2 (29)	3 (43)
Diarrhea	2 (29)	2 (29)	0 (0)	0 (0)
Fatigue	4 (57)	4 (57)	0 (0)	0 (0)
Hypocalcemia	2 (29)	2 (29)	0 (0)	0 (0)
Hypophosphatemia	2 (29)	1 (14)	1 (14)	0 (0)
Dyspnea	2 (29)	2 (29)	0 (0)	0 (0)

Adverse events occurring in >1 patient (*n* = 7).

Median time to maximum grade of all cytopenias was 36 days (0–64 days). Median time to resolution (grade 2 or less) of any cytopenia was 21 days (0–42 days) after nadir. Among patients experiencing thrombocytopenia, the median time to nadir was 36 days (29–50 days) and a median of 21 days (14–41 days) to resolution post nadir. Median time to maximum grade for patients having any grade neutropenia was 43 days (0–64 days) with a median of 7 days (7–28 days) to resolution after reaching maximum grade.

Initial findings from this CLOVER-1 expansion cohort of quad-class refractory (including anti-BCMA immunotherapy) R/RMM patients adds to the body of evidence that fractionated dosing achieving ≥60 mCi total body dose of iopofosine can be safely administered, leading to an ORR of 50%, with stable disease or better for all treated patients. The depth and durability of response to iopofosine in this heavily pretreated, refractory population compares favorably with previous experience with iopofosine [8, 9] and other treatments being used alone in similar patient populations [11–13]. One limitation of this primary analysis was its small patient cohort and the short duration of follow-up. Continued patient enrollment in this cohort as well as a longer follow-up is needed.

Several BCMA-directed therapies have been approved or are in clinical development, including CAR T-cell therapies, bispecific antibodies, and antibody drug conjugates [2]. CAR T therapy studies have shown a high number of patients achieving response; however, several challenges make this option unsuitable for R/RMM patients who are unfit for the conditioning regimens or who have inadequate disease control [4, 14]. Although the response rates for the CAR Ts range from 70 to >90%, high relapse rates (50%) within the first 12 months continue to pose clinical challenges. Additionally, tolerability remains a concern with high rates of cytokine release syndrome and neurotoxicity [2]. Another anti-BCMA immunotherapy alternative, belantamab mafodotin (an anti-BCMA antibody drug conjugate using the microtubule-targeting cytotoxin monomethyl auristatin-F), was recently approved by the FDA for treatment of R/RMM patients who have received ≥4 prior therapies (including anti-CD38 antibodies, PIs, and IMiDs) [2]. Similar to the CAR T therapies, early relapse and resistance to belantamab remain a challenge, leaving post anti-BCMA an area of unmet need in MM [8]. As increased use of the anti-BCMA immunotherapies is anticipated, this patient population is expected to increase and need treatment options. As demonstrated in a recent publication, 7 patients were evaluated for ORR for the first sequential antimyeloma therapy (sAMT) following idecabtagene vicleucel which was 28.5% (2 of 7) [6]. The clinical benefit rate (defined as stable disease or better) was 57.1%. The median PFS following 1st post-CAR T sAMT was 2 months (95% CI: 0–NR). Thus, investigation and development of novel agents with new mechanism of action such as iopofosine is highly desirable.

Iopofosine shows promising antimyeloma activity with four doses over 70 days in patients with heavily pretreated disease (median 9 lines of prior therapy) who are triple-class refractory and refractory or relapsed to anti-BCMA immunotherapy. This finding contributes to the growing body of evidence that iopofosine may be applicable to a complicated and diverse R/RMM population. Iopofosine’s novel mechanism of action, manageable safety profile, and limited number of treatments needed make it a potential candidate for use in combination treatment regimens. Ongoing (NCT02952508) and planned trials will investigate iopofosine combinations with new and standard-of-care treatments.

## Data Availability

Original data and protocols are available to other investigators upon request.
